# The growing need to integrate digital mental health into psychiatric and medical education

**DOI:** 10.1192/j.eurpsy.2024.1802

**Published:** 2024-12-27

**Authors:** Jorge Lopez-Castroman, Antonio Artés-Rodríguez, Philippe Courtet, Cecile Hanon, Tomasz Gondek, Enrique Baca-García, Umberto Volpe

**Affiliations:** 1 Department of Psychiatry, Radiology, Public Health, Nursing and Medicine, University of Santiago de Compostela, Santiago de Compostela, Spain; 2 Institut de Génomique Fonctionnelle, University of Montpellier, CNRS-INSERM, Montpellier, France; 3 CIBERSAM, ISCIII, Madrid, Spain; 4 Department of Psychiatry, Nimes University Hospital, Nimes, France; 5 Department of Signal Theory and Communications, Universidad Carlos III de Madrid, Madrid, Spain; 6 Instituto de Investigación Sanitaria Gregorio Marañón, Madrid, Spain; 7 Department of Emergency Psychiatry and Acute Care, CHU Montpellier, Montpellier, France; 8 Regional Resource Center of Old Age Psychiatry, AP-HP, Centre – University of Paris, Paris, France; 9 Psychiatry and Addictology, Corentin-Celton Hospital, Issy-les-Moulineaux, France; 10 Institute of Social Studies, University of Lower Silesia, Wroclaw, Poland; 11 Department of Psychiatry, University Hospital Jimenez Diaz Foundation, Madrid, Spain; 12 Health Research Institute IIS Jimenez Diaz Foundation, Madrid, Spain; 13 Madrid Autonomous University, Madrid, Spain; 14 Department of Psychiatry, University Hospital Villalba, Madrid; 15 Department of Psychiatry, University Hospital Infanta Elena, Valdemoro, Madrid; 16 Department of Psychiatry, University Hospital Rey Juan Carlos, Mostoles, Madrid, Spain; 17 Universidad Católica del Maule, Talca, Chile; 18 Unit of Clinical Psychiatry, Department of Clinical Neurosciences/DIMSC, School of Medicine, Università Politecnica delle Marche, Ancona, Italy

**Keywords:** Psychiatric Education, Medical Training, Mental Health Technologies, Healthcare Innovation, Digital Therapeutic

## Viewpoint

Incidence and burden of mental illnesses have increased over recent decades, with major depression, accounting for 1 in 6 years lived with disability [1]. The COVID-19 pandemic has revealed a global mental health epidemic that had been existing silently for years witnessing a crisis in public mental health systems across Europe. Prior to the pandemic, over 84 million individuals (approximately one in six people) were affected by mental health problems in the European Union, with a notable impact on the younger population [2]. Not surprisingly, European health systems are actively implementing policies to enhance access to care, improve working conditions for mental health workers, and increase the overall quality of mental health care [3]. The World Health Organization (WHO) also highlights the urgent need to diversify and scale up care options for common mental health conditions, as well as using digital technologies to support guided and unguided self-help and deliver remote care [1]. Indeed, digital applications provide a pathway to partially address global mental health challenges, particularly for the digitally native youth.

Digital resources offer powerful solutions to address population needs in five key areas: secondary prevention through screening, enhancing access to affordable health care, personalizing in-person care (including early intervention and prevention), improving self-care and self-management, and bridging the gap between healthcare demand and supply. The rapid advancement of Digital Mental Health (DMH) tools is evident as psychiatric and psychology departments worldwide spearhead the creation of new platforms for therapy delivery, resource provision, and the identification and monitoring of at-risk patients. Some digital tools, such as telemental health care, have been clinically available for more than a decade and are supported by robust evidence [4]. Meanwhile, other digital tools are rapidly gaining consensus and adoption within the field. M-health tools, including mobile apps and wearable devices, can collect and analyze user data to detect early signs of distress. Machine learning algorithms can identify patterns in user behavior, emotions, and engagement, trigger proactive interventions, or provide personalized coping strategies. Soon, cross-disciplinary approaches combining psychiatric and data-science expertise are expected to assist in the prevention of behavioral risks at an individual, personalized level.

Most researchers, stakeholders, and mental health professionals, and maybe even to a larger degree the public that is already using mental health apps widely, acknowledge the present and future potential of DMH. Nevertheless, this has exerted, so far, little impact on the educational programs of health students in most European countries. It could be argued that some of these tools are not yet ready for wide implementation in clinical practice, that their modes of use may change in the future, preempting the interest of addressing the educational needs for the moment, and that user engagement on digital therapies tends to decline over time due to factors like poor design and privacy concerns [5]. In this paper, we argue the reasons to start preparing health students on the use of DMH, which include:

- The adoption of DMH is currently underway, albeit predominantly driven by private companies vying to develop new products and capitalize on commercial opportunities. There are tens of thousands of mental health and wellness apps available, but very few have undergone rigorous testing [6]. Medical students should be equipped to guide patients toward validated and reliable apps. To achieve this, they need to understand the potential benefits and risks these apps present to patients.
- There exists a notable time gap between medical education and independent professional practice. Considering the rapid pace of technological advances and accumulating experience, DMH will very probably be routinely used by many patients over the upcoming years.
- Training in DMH should not be limited to future psychiatrists. Considering the pervasive impact of mental health issues across medical specialties, training should be accessible to all health professionals, particularly to general practitioners, by far the main collaborators of psychiatrists in the treatment of psychiatric disorders, as well as neurologists or internal medicine physicians.
- The expanding necessities in mental health care are likely to aggravate the limited availability of specialized professionals. According to the WHO, the number of mental health workers fell from 50 per 100,000 population in 2017 to less than 45 in 2020 in the European region. Training in DMH could increase awareness and understanding across specialties regarding the management of common mental disorders while also mitigating biases.
- Digital education facilitates early adoption and reduces stress, frustration, and avoidance of digital tools. The human factor, both in professionals and patients, is essential for the uptake of digital therapeutics in mental health [7]. A better training at the undergraduate stage could lead to more healthcare professionals becoming actively involved in the development of digital therapeutics, thus improving their quality. DMH can also help to enhance health literacy in the general population and reduce the stigma associated with mental disorders through increased accessibility and education.
- User engagement in digital therapies can be significantly enhanced by incorporating human support, gamification, contingency management, and interactive features [5]. Advances in technology and cultural shifts toward digital solutions offer tremendous potential to make these therapies more appealing and effective. They can help build healthy habits in various areas of life, positively impacting well-being.

We do not advocate for increasing the burden on medical education, but it seems essential to adapt its methods and contents to align with advancing technology. DMH could be integrated within digital health courses as a specific focus. Undergraduate training in digital health is expanding, but it often overlooks mental health requirements. The profound implications for mental health stem from its significant burden, rising prevalence, and shortage of professionals for common mental disorders. The vast array of DMH tools promises streamlined, personalized patient care. Leading mental health apps today offer psychoeducation, therapy techniques, mood monitoring, self-assessment, and community support [8]. Recent treatment guidelines recommend digital therapeutics, acknowledging current tool limitations. For example, the latest CANMAT guidelines endorse guided DMH as a first-line treatment for mild to moderate depression [6]. These therapies require clinical training for professionals who support users and must be cost-effective and adaptable to ensure accessibility across various cultures and income levels. Despite widespread integration in developed countries, Europe shows a heterogeneous landscape regarding reimbursement [9].

Among the generic professional profiles that can be delineated within the digital health competency framework [10], community health and care coordination are particularly relevant for DMH. In particular, the specific educational statements about DMH are:

- Evaluating the validity and usefulness of DMH tools involves assessing their efficacy, efficiency, privacy/security, risks, and access [6].
- DMH can improve access to quality care and optimize the use of existing resources, thereby reducing costs [1].
- The specific components of telehealth that enhance its efficacy and efficiency in mental health care include diagnostic reliability, therapeutic alliance, accessibility, acceptability, and improved patient outcomes [4].
- Handling real-time inputs and monitoring information in patient follow-up requires improving engagement, managing psychological crises and treatment side effects, and fostering the physician-patient relationship through both synchronous and asynchronous interactions.
- Models for Clinical Decision Support Systems in mental health, including Just-In-Time Adaptive Interventions based on real-time data from wearables, should address ethical issues related to patient autonomy, stigma, digital exclusion (e.g., when caused by digital illiteracy or economic factors such as lack of access to high-end mobile devices), privacy, and data confidentiality.
- DMH can be used for public health interventions including secondary prevention through screening, online support communities and forums, peer support networks, discussion groups, educational campaigns, and interactive psychoeducational modules.
- Healthcare professionals should be actively involved in the design and development of DMH tools. Some will need to acquire the skills to interpret data, develop programming capabilities, and interact with artificial intelligence systems. Healthcare students should recognize that engaging with these emerging technologies presents a viable and promising career path.

To conclude, DMH tools already offer scalable and flexible solutions that improve access to care and support various aspects of mental health provision. As their use becomes more prevalent, health students must understand how these tools work and be aware of their benefits and risks. DMH training would prepare students to critically evaluate these tools and shape informed opinions in an era where DMH solutions are flooding the market. Integrating DMH in educational programs is essential and should be part of both graduate and undergraduate medical and psychiatric education.
